# Multimodal Retinal Image Analysis via Deep Learning for the Diagnosis of Intermediate Dry Age-Related Macular Degeneration: A Feasibility Study

**DOI:** 10.1155/2020/7493419

**Published:** 2020-01-13

**Authors:** Ehsan Vaghefi, Sophie Hill, Hannah M. Kersten, David Squirrell

**Affiliations:** ^1^School of Optometry and Vision Sciences, University of Auckland, Auckland, New Zealand; ^2^Auckland Bioengineering Institute, University of Auckland, Auckland, New Zealand; ^3^Department of Ophthalmology, University of Auckland, Auckland, New Zealand

## Abstract

**Results:**

The CNN trained using OCT alone showed a diagnostic accuracy of 94%, whilst the OCT-A trained CNN resulted in an accuracy of 91%. When multiple modalities were combined, the CNN accuracy increased to 96% in the AMD cohort.

**Conclusions:**

Here we demonstrate that superior diagnostic accuracy can be achieved when deep learning is combined with multimodal image analysis.

## 1. Introduction

In the developed world, age-related macular degeneration (AMD) is the leading cause of irreversible vision loss in the population over 60 years old [1, 2]. Broadly AMD can be categorised in neovascular and non-neovascular AMD, with the later being far more prevalent. Characteristic features of non-neovascular AMD include macular drusen, RPE abnormalities, and, in the late stage, geographic atrophy [3]. Pigment abnormalities detected by colour fundus photography (CFP) are now well recognised to be one of the major risk factors for the development of late stage AMD [3–6].

Over time, optical coherence tomography (OCT) and fundus autofluorescence (FAF) have also joined the battery of imaging techniques that are now considered essential for the monitoring of non-neovascular AMD. This list has more recently been joined by optical coherence tomography angiography (OCT-A) and multicolour confocal scanning laser ophthalmoscopy (SLO) [7]. With the introduction of OCT and OCT-A guided AMD treatment regimens, there has been an exponential increase in the number of images obtained and stored in large electronic databases [8]. Furthermore, the multiple imaging techniques that are now routinely used in clinical practice mean that the cumulative time spent by retinal specialists interpreting these images is also increasing [8]. Utilising software or computation power to automate image analysis may have the potential to improve the efficiency and accuracy of this process in the clinic [9]. Computer-assisted image assessment is also less prone to human factors such as bias, fatigue, and mindset [10]. Computer-assisted diagnosis is not a new concept: radiology adopted this approach when the increasing demand for imaging studies outstripped the ability of radiologists to interpret and report on the studies [11].

Within the field of ophthalmology, automated image analysis has been applied to the detection of diabetic retinopathy, mapping visual field defects in glaucoma, and grading cataract [12–14]. Concentrating on macular disease, both semiautomated and automated techniques have already been used and validated in the detection of drusen, reticular drusen, and geographic atrophy [15–19]. The majority of these studies have developed networks using only a single imaging modality, namely, OCT [20–24], and very few have combined image data from more than one modality, e.g., CFP and OCT images [25], or infrared, green FAF, and SLO [26].

The purpose of this study is to determine whether a multimodal deep learning approach; training the CNN on OCT, OCT-A, and CFP, will diagnose intermediate dry AMD more accurately, when compared to conventional CNN trained on the single modalities of CFP, OCT, and OCT-A.

## 2. Methods

Seventy-five participants were recruited through Auckland Optometry Clinic and Milford Eye Clinic, Auckland, New Zealand. All participants provided written informed consent prior to imaging. Ethics approval (#018241) from the University of Auckland Human Participants Ethics Committee was obtained for this study. This research adhered to the tenets of the Declaration of Helsinki. Participants were divided into three groups: young healthy (YH), old healthy, (OH) and high-risk intermediate AMD (AMD). A total of 20 participants were recruited into the YH group, 21 into the OH group, and 34 into the AMD group. A comprehensive eye exam was conducted on each participant prior to the OCT and OCT-A scans including dilated fundal examination and high contrast best-corrected visual acuity (BCVA) to determine the ocular health status of the fundus. Patients with any posterior eye disease that could potentially affect the choroidal or retinal vasculature including but not limited to glaucoma, polypoidal choroidal vasculopathy, DR, hypertensive retinopathy, and high myopia (≥6 D) were excluded from the study. Patients with any history of neurological disorders were also excluded. None of our recruited participants fit the exclusion criteria. The YH group consisted entirely of individuals between the ages of 20 and 26 and a best corrected visual acuity of ≥6/9 in the eye under test. The OH group consisted of individuals over the age of 55 years who had a best corrected visual acuity of ≥6/9 in the eye under test and a normal ocular examination. The “AMD cohort” consisted entirely of patients with high-risk intermediate dry AMD. This was diagnosed if the individual had at least two of the following risk factors: reticular pseudodrusen, established neovascular AMD in the fellow eye, and confluent soft drusen with accompanying changes within the retinal pigment epithelium. In order to ensure that all patients in the “AMD cohort” could maintain fixation during OCT-A imaging, only those patients with a BCVA of 6/15 or better were enrolled. The mean age of the participants in the YH, OH, and AMD groups were 23 ± 3, 65 ± 10, and 75 ± 8 years, respectively. Only one eye of each patient was used for the analysis, and if the patient had both eyes scanned, the OCT-A scan that had the better quality (assessed subjectively by the clinical grader) of the two was used. Mean BCVA for the YH, OH, and AMD groups were 6/6, 6/9, and 6/12, respectively. The ocular health of all participants was assessed at Auckland Optometry Clinic, by a registered optometrist, prior to enrolment in the study. The macular status of patients enrolled into the AMD group was assessed separately by an experienced retinal specialist (DS).

### 2.1. SS-OCT-A Device and Scanning Protocol

Participants were dilated with 1.0% tropicamide if the pupils were deemed too small for adequate OCT scans. The swept source (SS) OCT-A device (Topcon DRI OCT Triton, Topcon Corporation, Tokyo, Japan) was used to obtain the following images: 3 × 3 mm^2^ macular *en-face* OCT and 3 × 3 mm^2^ macular *en-face* OCT-A.

**Figure 1:**
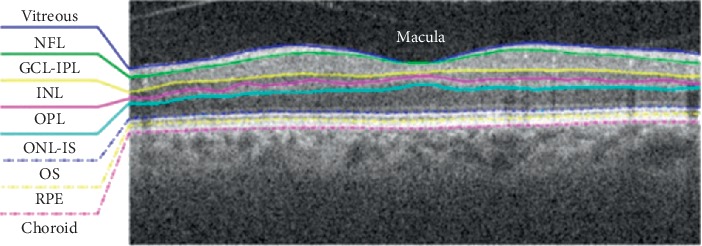
The retinal layers automatically identified by IMAGEnet 6.0 software. NFL: nerve fiber layer; GCL: ganglion cell layer; IPL: inner plexiform layer; INL: inner nuclear layer; OPL: outer plexiform layer; ONL: outer nuclear layer; IS: inner segment; OS: outer segment; RPE: retinal pigment epithelium.

**Figure 2:**
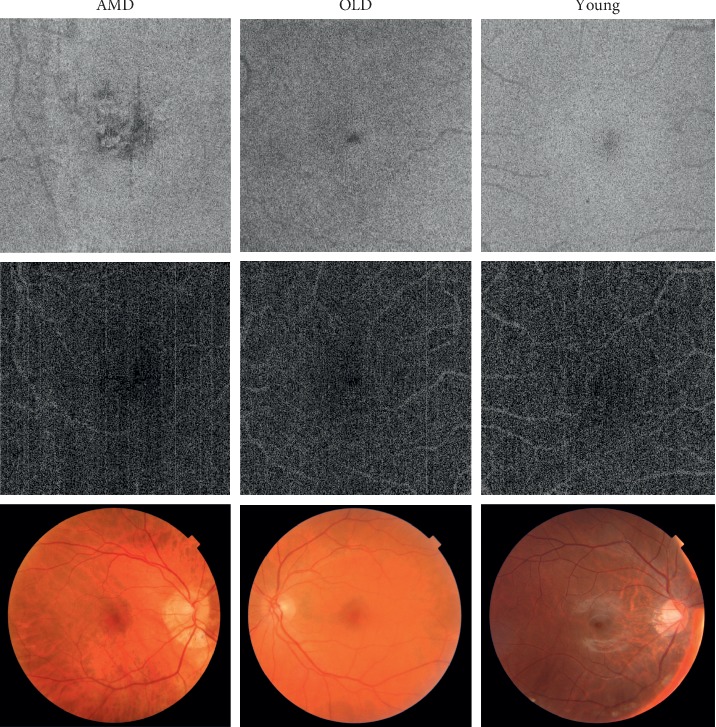
Examples of *en-face* OCT (top row), *en-face* OCT-A (middle row), and colour fundus photographs (bottom row) from each of the three cohorts (AMD, OH, and YH). The images within each column are from the same patient, from the labeled cohort at the top.

Raw OCT, OCT-A, and CFP image data were exported using Topcon IMAGEnet 6.0 software. No image processing was performed prior to image analysis. The retinal layers were identified using the IMAGENET 6.0 automated layer detection tool (Figure 1). *En-face* OCT and OCT-A images from layers 6 to 9 of the scan were selected (ONL-IS, OS, RPE, and choroid), plus a single fundus photo. The dataset was divided into 0.6/0.2/0.2 for training, validation, and test sets. This division was based on participants (not images) in order to avoid data leakage between the training, validation, and test sets. As there were multiple OCT and OCT-A images per patient, appropriate measures were taken to ensure that there was no patient overlap between the training, validation, or test sets (Figure 2).

### 2.2. Convolutional Neural Network Design

**Figure 3:**
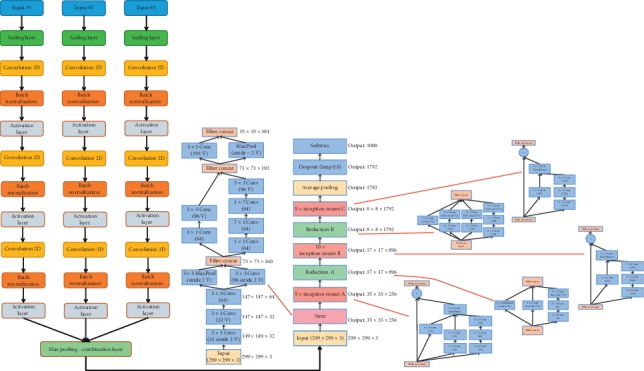
Modification to the top layers of INCEPTION-RESNET-V2 architecture.

The original design of the convolutional neural network (CNN) used here was based on the INCEPTION-RESNET-V2 design, since it appeared to have the advantage of faster convergence speed. This design was further modified to enable the network to be trained on multiple image modalities at the same time (Figure 3). Each imaging stream was then set up to initiate with a resizing layer, which was then followed by three repetitions of a 2D convolutional layer, batch normalization, and RELU activation layer. Separate modalities were then concatenated using a global pooling layer. The main body of the CNN followed the classic INCEPTION-RESNET-V2 design, where blocks of inception cells (A, B, and C) were used in series, where each block included cells with varying kernel sizes and channels, as well as feed forwards bypasses. The Python code for creating the CNN is released and freely available here: https://medium.com/@mannasiladittya/building-inception-resnet-v2-in-keras-from-scratch-a3546c4d93f0.

Experiments were run on an Intel Xeon Gold 6128 CPU @ 3.40 GHz with 16 GB of RAM memory and a NVIDIA GeForce TiTan V VOLTA 12 GB, for 100 epochs. The training-stop criterion was as CNN validation loss (measured as negative log-likelihood and residual sum of squares) had reached a stable minimum over the last 3 EPOCHs of training. Hence, any further training would have led to model “overfitting,” in which the neural network “memorizes” the training examples. It is worth noting that this approach is scalable to include more modalities (>3).

Several network training episodes were undertaken. Firstly, only a single image modality was used: OCT, OCT-A, or CFP. Further training episodes combined both OCT and OCT-A image data. The last training episode utilised multimodal image analysis from OCT, OCT-A, and CFP.

To better understand the nature of “learning” from our CNN in each modality, attention maps were produced from each modality. This method is explained in detail elsewhere [27–29]. Briefly, the output of the last convolutional layer prior to the global concatenation was extracted, resized, and smoothed for visualization.

## 3. Results

Table 1 shows that the sensitivity and specificity of the CNNs trained using a single modality are high. However, when more than one image modality is used during the training of the CNN, the sensitivity and specificity increase with each additional image modality added. The CNN with the highest accuracy, sensitivity, and specificity was the multimodal image-trained network.

If the CNN results are considered for each imaging modality, it would suggest that each modality is better suited for classifying certain categories. The CFP single modality CNN was the most sensitive to AMD closely followed by OCT-A. In contrast, the single modality OCT was more sensitive to ageing, identifying the young and old cohorts more accurately. Combining the imaging modalities into a single “multimodal” CNN resulted in an accuracy of 99.8%, being able to identify both ageing and disease with high sensitivity and specificity (Table 1).

**Figure 4:**
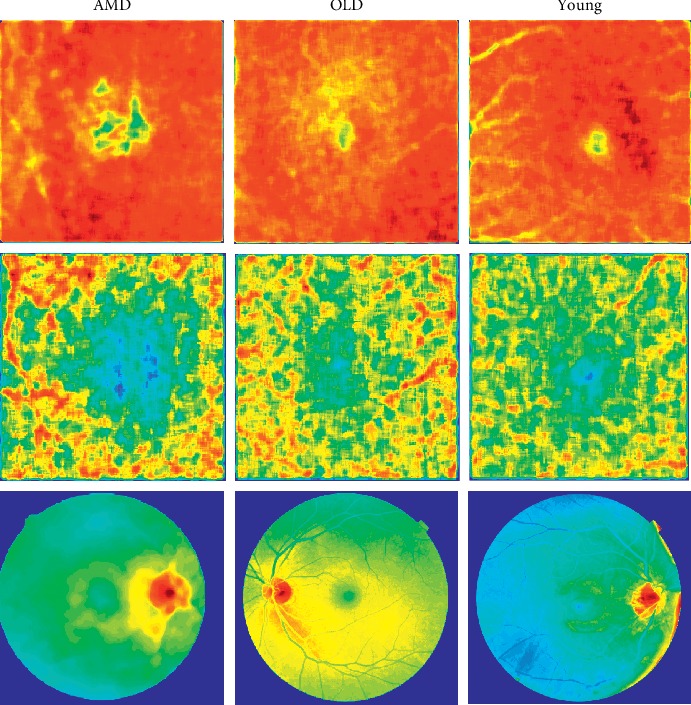
Same images as Figure 2 were used to generate these attention maps. Normalized images [0-1] are pseudocoloured based on the level of “attention” the CNN gave to the region in the image modality (low represented by blue, to high represented by red).

To investigate the apparent different sensitivities for each modality, “attention maps” of each modality were generated (Figure 4) from the same images in Figure 2. These maps demonstrate the image features that were “noticed” by multiple CNNs for each modality or participant group.

The attention maps of the OCT images in each cohort pay highest attention to the background homogeneity. In the OCT-A images, the areas of highest attention are what appear to be projection artefacts of the retinal vessels seen within the choricapillaris OCTA slab, and the surrounding background OCTA signal of the adjacent tissues. In CFP, the area of highest attention is at the optic disc and the peripapillary region in young and AMD and additionally at the macula in the old cohort.

## 4. Discussion

In the current study, we wanted to compare the accuracy of different CNN designs, trained on the same dataset, to investigate whether combing imaging modalities improved the ability of the CNN to accurately identify the 3 distinct clinical groups under test. A secondary aim was to evaluate the attention maps of each design to investigate which components of the image(s), the CNN “paid attention” to.

To date, a number of single image modality CNNs designed to identify AMD have been published, with differing rates of success [30–36]. Furthermore, the majority of these studies have been based on single transverse OCT image (cross section) taken through the fovea [23, 37–40]. Although such an approach, training a CNN on a single OCT image, can achieve impressive results, this approach is flawed as a single transverse image will only sample a very limited part of the macula and relies on the pathology being present within the scan analysed.

The use of *en-face* OCT images may overcome the segmentation error and sampling bias associated with the use of transverse OCT scans. This technique has previously been utilised by Zhao et al. [33], to automatically detect the presence of drusen, including pseudodrusen. CFPs have also been used as a single imaging modality in the identification of AMD using an appropriately trained CNN [30, 32, 34–36, 41]. Good levels of accuracy have been obtained [30, 34], but again often only after image resizing and significant image preprocessing [36].

To the best of our knowledge, this is the first study to develop a CNN that utilises both *en-face* OCT and OCT-A and then combines them with CFP to develop a truly “multimodal” CNN, one that truly samples the entirety of the macular under test. To avoid sampling bias, the *en-face* OCT and OCT-A data slabs of the entire macula were used. We found that the multimodal CNN was superior to the single modality CNNs; moreover, incorporating additional modalities led to an incremental improved accuracy of the CNN.

To the best of our knowledge, only two other groups have utilised a similar multimodal approach [25, 42]. Yoo et al. [25] used paired fundus and OCT images, employing a VGG19 model pretrained on ImageNet to extract visual features from both images. More impressive is the approach taken by Wang et al. [42], who used a training method they termed “loose pairing” whereby pairings were constructed based on labels instead of eyes. In this method, a fundus image is allowed to be paired with an OCT image if their labels are identical, an approach which yielded an overall accuracy of 97%. The progressive improvement in the performance of the incrementally complex CNNs that Wang et al. [42] and ourselves describe strongly suggest that, like clinicians, the accuracy of a CNN to detect pathology and “normal” ageing is enhanced if complimentary imaging modalities are employed.

The outcome of our single modality CNNs (Table 1) suggest that OCT and OCT-A modalities are inherently sensitive to different aspects of the retinal health. It appears that OCT is more sensitive to ageing of the retina, while CFP and OCT-A are better suited to detect the pathological changes attributable to AMD. The black box nature of the neural networks makes any interpretation of the results problematic [43]. We therefore produced attention maps to aid our understanding of the CNN activity. The *en-face* OCT images in each cohort revealed that the highest attention was directed to the background homogeneity with a lesser emphasis on the fovea. OCT-A in contrast appears to be more sensitive to disease. Review of the attention maps reveals that within the OCT-A images, the regions of higher attention are the retinal vessels and the tissues immediately adjacent to them. Again the fovea appears to contribute very little to the OCT-A CNN classifier.

In conclusion, although trained on a small number of images, this study demonstrates that, compared with CNNs trained on a single image modality, superior diagnostic accuracy can be achieved when deep learning is combined with multimodal image analysis. We should also emphasise that this is only a “proof of concept” study, and larger studies to test its validity are needed. It should also be noted that the attention maps produced here are from our small cohort and may not prove to be generalisable after further studies. This approach is more similar to the multimodal image interpretation used by retinal specialists within the clinical environment, and our results suggest that this approach warrants further investigation. The limitations of this study include the small sample size of images, with the CNN trained using images from a single academic center. These clearly limit the generalisability of the CNNs we have trained, but arguably do not detract from the conclusion that a multimodal CNN is superior to a single modality CNN. Further research would include the utilisation of images from other OCT manufacturers, and validation of this CNN on a dataset from another academic or clinical institution.

## Figures and Tables

**Table 1:** The accuracy, sensitivity, and specificity of each CNN design (single modality vs. dual modality vs. multimodality) in classification of three cohorts of YH, OH, and AMD.

	Patient cohort		
	YH	OH	AMD
Single modality CNN	Overall accuracy 94.4%		
OCT sensitivity (%)	99.6	98.9	77.8
OCT specificity (%)	98.8	86.7	100
Single modality CNN	Overall accuracy 91.9%		
OCT-A sensitivity (%)	95.5	83.2	97.6
OCT-A specificity (%)	99.6	96.2	76.4
Single modality CNN	Overall accuracy 93.8%		
CFP sensitivity (%)	81.0	84.6	100
CFP specificity (%)	94.4	78.6	86.7
Dual modality CNN (OCT + OCT-A)	Overall accuracy 96.7%		
Sensitivity (%)	100	95.7	92.1
Specificity (%)	97.6	94.6	98.7
Dual modality CNN (OCT + CFP)	Overall accuracy 92.9%		
Sensitivity (%)	98.1	96.3	100
Specificity (%)	100	97.0	96.0
Multimodality CNN (OCT + OCT-A + CFP)	Overall accuracy 99.8%		
Sensitivity (%)	100	99.3	100
Specificity (%)	100	100	99.2

## Data Availability

The ophthalmic imaging data used to support the findings of this study were obtained under appropriate approval by the University of Auckland Ethics Committee and so cannot be made freely available. Requests for access to these data should be made to the corresponding author Dr Ehsan Vaghefi e.vaghefi@auckland.ac.nz, which will be then passed on to the University of Auckland Ethics Committee for further process.
